# Selective extraction of recombinant membrane proteins from *Hansenula polymorpha* by pulsed electric field and lytic enzyme pretreatment

**DOI:** 10.1186/s12934-023-02259-z

**Published:** 2023-12-08

**Authors:** Valentina Ganeva, Andreas Kranz

**Affiliations:** 1 Biological Faculty, Department of Biophysics & Radiobiology, Sofia University, 8 Dragan Tzankov blvd, Sofia, 1164 Bulgaria; 2 ARTES Biotechnology GmbH, Elizabeth Selbert str. 9, 40764 Langenfeld, Germany

**Keywords:** *Hansenula polymorpha*, Pulsed electric field treatment, Flow system, Lytic enzyme, Recombinant membrane proteins, Virus like particles

## Abstract

**Background:**

In yeast, recombinant membrane proteins including viral scaffold proteins used for the formation of enveloped Virus-like particles (eVLPs) typically accumulate intracellularly. Their recovery is carried out by mechanical disruption of the cells, often in combination with detergent treatment. Cell permeabilization is an attractive alternative to mechanical lysis because it allows for milder and more selective recovery of different intracellular products.

**Results:**

Here, we present a novel approach for extraction of integral membrane proteins from yeast based on cell envelope permeabilization through a combination of pulsed electric field and lytic enzyme pretreatment of the cells. Our primary experiments focused on *Hansenula polymorpha* strain #25-5 co-expressing the integral membrane small surface protein (dS) of the duck hepatitis B virus and a fusion protein of dS with a trimer of a Human papillomavirus (HPV) L2-peptide (3xL2-dS). Irreversible plasma membrane permeabilization was induced by treating the cell suspension with monopolar rectangular pulses using a continuous flow system. The permeabilized cells were incubated with lyticase and dithiothreitol. This treatment increased the cell wall permeability, resulting in the release of over 50% of the soluble host proteins without causing significant cell lysis. The subsequent incubation with Triton X-100 resulted in the solubilization and release of a significant portion of 3xL2-dS and dS from the cells. By applying two steps: (i) brief heating of the cells before detergent treatment, and (ii) incubation of the extracts with KSCN, an 80% purity on the protein level has been achieved. Experiments performed with *H. polymorpha* strain T#3-3, co-expressing dS and the fusion protein EDIIIWNV-dS consisting of dS and the antigen from the West Nile virus (WSV), confirmed the applicability of this approach for recovering dS. The treatment, optimal for solubilization of 3xL2-dS and a significant part of dS, was not effective in isolating the fused protein EDIIIWNV-dS from the membranes, resulting in its retention within the cells.

**Conclusions:**

This study presents an alternative approach for the recovery and partial purification of viral membrane proteins expressed in *H. polymorpha*. The factors influencing the effectiveness of this procedure and its potential use for the recovery of other integral membrane proteins are discussed.

## Background

Virus-like particles (VLPs) are multiprotein nano-size structures that mimic the organization and conformation of native viruses but lack the viral genome and thus are non-infections. They are formed on the basis of viral proteins from the nucleocapsid or the virus envelope which have the ability to self-assembly and form non-enveloped VLPs and enveloped VLPs, respectively [1, 2].

Nowadays VLPs formed from recombinant viral proteins expressed in different host systems find a number of applications, first of all as novel vaccines (VLP-based vaccines), but also as a drug, nucleic acid and protein delivery systems, as well as for fundamental research in virus-host, protein-protein and protein-lipid interaction studies [3–5].

Currently, yeasts are among the most widely used systems for the production of VLPs for various purposes. They offer several advantages, including rapid cell growth, high yields of the expressed proteins, cost-effective production, and biosafety benefits over other microbial systems due to their lack of endotoxin production [3, 6].

Over the last 30 years, the methylotrophic yeast *Hansenula polymorpha* (syn. *Ogataea polymoprha*) has been intensively used as a host for commercial production of different recombinant proteins including both enveloped (eVLPs) and non-enveloped virus-like particles (VLPs) [7–10]. It offers very high expression rates that is linked to utilisation of strong, carbon source-regulated promotors of the *MOX* and *FMD* genes, high copy numbers of the expession cassettes and mitotic stability of integrated plasmids [11, 12]. Another impоrtant aspects are the organism’s ability to grow to high cell densities on simple, inexpesnive media, as well as its thermotolerance.

The process of VLP formation certainly depends on the scaffold protein used, and details of these processes have not yet been fully elucidated. In some examples, the viral proteins expressed in yeast are either initially bound at the ER membrane or forme multi-layered lamellar structures localized into the lumen of the ER, so the VLPs formation (assembly) and maturation accures in vitro, during the different stages of the purification process [13–16]. Other publications show that the viral proteins expressed can form VLPs in vivo, that accumulate in the cytosol or are retained in the periplasm due to the limited cell wall porosity [17–20]. Moreover, examples for secretory production of VLPs by yeast were reported; however, the yields obtained so far are low [21].

Generally in yeast, viral scaffold proteins used for formation of enveloped VLPs tend to accumulate as an intracellular product, and their recovery is carried out by mechanical disruption of the cells, most often in combination with detergent treatment [10, 13–16].

The mechanical cell lysis, although widely used in bioindustry, leads to non-selective release of intracellular components, increase of the viscosity and micronization of the cell debris, which complicates the clarification of the cell lysates by centrifugation and the following downstream processing [22]. Hence, host cell protein depletion remains one of the most complex steps in the purification of VLPs [6, 14, 15] Thus, development and application of a scalable method for more selective release of VLPs or VLPs forming recombinant proteins is very attractive, as this could render the VLP manufacturing easier and significantly cheaper.

Various approaches have been taken to improve selectivity. A recent study conducted by Kee et al. [14] has shown that recovery and primary purification of eVLPs can be enhanced by utilizing moderate homogenization pressure conditions to reduce cell fragmentation and performing a centrifugation step before the addition of detergent.

Another approach for more selective release of recombinant proteins (including VLPs) expressed in yeast is the partial removal of the cell wall with lytic enzymes, yielding spheroplasts. Asenjo et al. [23] demonstrated liberation of VLPs localized in the cytosol of *S. cerevisiae*, after preparation of spheroplasts with pure glucanases and their subsequent treatment with Triton X- 100. Shen et al. [20] found that incubation of *H. polymorha* cells with glucanase in osmotically balanced medium, leads to the release of 22 nm particles of preS2-HBsAg, which were secreted but retained in the periplasm. Sakuragi et al. [24] demonstrated continuous HIV type 1 Gag virus-like particle buding from the plasma membrane of *S. cerevisiae* spheroplasts. However, commercial use of this method does not seem attractive because of the high cost of lytic enzymes.

During the last few years, a lot of data has been collected on the application of pulsed electric field (PEF) treatment as a selective, non-thermal method for extraction of bioactive compounds from microorganisms and plant tissues [25, 26]. The electrical treatment provokes change in plasma membrane integrity known as electropermeabilization or electroporation [27, 28]. Dependant on the electrical parameters, nature and age of the cell used, the pulsing media composition, and post-pulse incubation, electropermeabilization can be either reversible or irreversible, and both selectivity and efficiency of protein release can be influenced [29–32].

A major advantage of moderate PEF treatment lies in the fact it does not provoke fragmentation of cells with cell walls in general [30–34]. Electropermeabilization leads to the release of smaller water-soluble components into the medium, whereas hydrophobic molecules and more complex structures like organelles and cell membranes, are retained inside unless an additional treatment, such as solvent addition or mechanical disruption, is applied [34–37]. This allows selective extraction of various intracellular components based on their size and charge, their location in different compartments and their hydrophobicity.

PEF treatment using a continuous flow system leads to the massive release of many native intracellular enzymes from different yeast species [33, 38]. It was shown that electrical treatment not only changes the integrity of the membrane, but additionally increases the porosity of the cell wall, thereby making late exponential and stationary phase cells more sensitive to lytic enzymes. This effect was also observed in experiments with a recombinant *S. cerevisiae* strain expressing *E. coli* β-galactosidase [39] and an *H. polymorpha* strain expressing and accumulating oligomers of the human ferritin heavy chain (FTH1-H6) in the cytosol [40]. By combining PEF treatment and subsequent incubation of the cells with a low concentration of lyticase, liberation and partial purification of these large soluble recombinant proteins was achieved. Recently, a similar procedure has been applied for the first time to enhance the extraction of recombinant membrane proteins from yeast [41].

This study presents an alternative approach for efficient and selective recovery of recombinant integral membrane proteins from *H. polymorpha*. It is based on cell envelope permeabilization through a combination of pulsed electric field and lytic enzyme pretreatment of the cells, followed by solubilization and extraction of the proteins with detergent. A VLP scaffold protein was selected as an example, namely the integral membrane small surface protein (dS) of the duck hepatitis B virus (DHBV), a novel promising platform for displaying antigens from different viruses on chimeric eVLPs [10]. This system allows incorporation of large antigens into the dS scaffold, which was successfully demonstrated with antigens from e.g. the bovine viral diarrhae virus (BVDV), classical swine fever virus (CSFV), west nile virus (WSV) or *Plasmodium falciparum* causing malaria disease.

Typically, VLP antigen incorporation and surface presentation is achieved by co-expression of dS and a fusion protein composed of the antigen genetically fused to dS in *Hansenula polymorpha*, leading to formation of chimeric VLPs [10, 42–44].

In the present study, experiments were performed with an *H. polymorpha* strain named “#25-5”, co-expressing dS and dS-based Human Papillomavirus (HPV) vaccine candidate. The permeability of the cell envelope (plasma membrane and cell wall) was increased by PEF treatment applied in a flow mode and a subsequent incubation of the cells with lytic enzyme and thiol compound. This treatment did neither result in a significant cell lysis, nor led to the release of the recombinant proteins, but allowed the removal of more than 50% of the host soluble proteins. Subsequent detergent treatment led to solubilization and release of a considerable portion of both recombinant proteins. Through application of two simple steps: *(i)* brief heating of the cells before detergent treatment, and *(ii)* incubation of the extracts with KSCN, an 80% purity at protein level has been obtained. Experiments performed with *H. polymorpha* strain T #3-3, co-expressing dS and the fusion protein EDIIIWNV-dS consisting of dS and the antigen from the West Nile virus (WSV), confirmed the applicability of this approach for recovering dS.

## Materials and methods

### Materials

Yeast extract and bacteriological peptone were purchased from Oxoid LTD (England), soy peptone, glycerol, glucose, acrylamide:bis-acrylamide from Thermo Fisher Scientific. Lyticase from *Arthrobacter luteus*, propidium iodide (PI), dithiothreitol (DTT), Triton X-100, Tween 20, phenylmethylsulfonyl fluoride (PMSF), Amberlite XAD4 and glass beads were obtained from Sigma-Aldrich. The anti-dS mouse monoclonal antibody (7C12) was obtained from BioGenes GmbH, Berlin, Germany and Alkaline Phosphatase - conjugated goat-anti-mouse antibody from Bio-Rad Laboratories, Inc.

### Yeast strain and culture conditions

The experiments were performed with *H. polymorpha* strain #25-5 (RB11/dS-3xL2-dS) co-expressing duck Hepatitis B S-antigen (dS) and a fusion protein consisting of dS and an HPV L2 antigen. For antigen presentation, a trimer of the L2 HPV16 (20–38) antigen [45] was genetically fused to the N-terminus of dS. Strain #25-5 is based on host strain RB11 (relevant genotype *ura3)* [46] derived from wild type strain ATCC 34,438 (CBS 47 32, IFO 1476, JCM 3621, NBRC 1476, NCYC 1457, NRRL Y-5445). Aiming co-expression of the two proteins, RB11 has been transformed with a dual expression plasmid as described by Wetzel et al. [10], coding for both proteins. The recombinant yeast cell line was generated by electroporation [47] and subsequent strain generation and isolation protocol. Thereby, the expression plasmids integrated genomically stable in different copy numbers into the host genome. Heterologous yeast strains were stored as glycerol stocks at − 80 ^o^C. Strain T#3-3 served as second example, co-expressing dS and a fusion protein EDIIIWNV-dS consisting of dS and the antigen from west nile virus (WSV) [10].

The cells were propagated in YPD medium (yeast extract-peptone-dextrose) at 37 ^o^C, 180 rpm for 16 h. For derepression 0.6 mL of cell suspension were used to inoculate 60 mL YPG (10 g L^− 1^ yeast extract, 20 g L^− 1^ soy peptone, 20 g L^− 1^ glycerol). The cultures were incubated in 300-mL flasks for 48 h at 37 ^o^C and 180 rpm in a Biosan ES-20/40 orbital shaker. For induction, to each flask methanol (final concentration 1% v/v) was added, and the cells were cultivated at 37 ^o^C for additional 24 h. After cultivation the cells were harvested by centrifugation (2600 x g for 10 min), washed twice and diluted in distilled water to a concentration corresponding to 90 mg wet cell weight mL^− 1^.

### Pulsed electric field (PEF) treatment

The electrical treatment was performed with a generator of monopolar rectangular pulses, a Hydropuls mini (GBS-Elektronik, Germany), in a continuous-flow chamber as previously described [32, 40]. The pulse duration and frequency were regulated by an arbitrary waveform generator RIGOL DG1012 (China). The chamber (0.3 mL volume) has two parallel stainless steel electrodes that are 0.3 cm, specially manufactured by MEHEL (Bulgaria). The PEF treatment was performed at flow rate of 25 mL min^–1^, controlled by a peristaltic pump. During the passage through the chamber, the cells received 30 pulses with a duration of 0.7 ms and electric field strength range of 5-6.5 kV cm^− 1^.

All pulsing parameters were monitored online with an Instek GDS 2064 oscilloscope (Taiwan). The inlet temperature of the suspensions was 23–24 ^o^C. The outlet temperature was registered by a K-type thermocouple connected to a digital thermometer, and the sensor was attached to the end of the tubing. Electrically treated and control cells were harvested by centrifugation at 2600 x g for 10 min at room temperature, diluted in 50 mM potassium phosphate buffer (PPB) pH 8 to a final concentration corresponding to 180 mg wet cell weight mL^− 1^ and stored at -20 ^o^C until further processing.

### Determination of irreversible electropermeabilization

The membrane permeabilization was assayed by loading the cells with propidium iodide (PI). To determine the fraction of cells with irreversibly permeabilized membranes, 5 µL of 0.5 mM solution of PI in distilled water were added to 20 µL cell suspension 1 h after pulsation of the cells. The number of fluorescent cells was counted under an epifluorescent microscope (L3201 LED, Microscopesmall, China). The permeabilization was expressed as a percentage of the number of fluorescent cells relative to the total cell number.

### Тreatment with lytic enzyme

The electropermeabilized and control untreated cells (suspension volumes 1-1.5 mL) were centrifuged at 10,750 x g for 1 min, and the cells were diluted in 125 mM PPB pH 8 to the same final concentration (180 mg wet cell weight mL^− 1^). PMSF was prepared at a concentration of 160 mM in isopropanol and added to the cell suspensions to a final concentration of 1.5 mM DTT (1 M stock solution) was added to final concentrations between 1 and 10 mM, and lyticase (stock solutions 3000–6000 U mL^− 1^) to final concentrations between 2.5 and 30 U mL^− 1^. The suspensions were incubated between 1 and 4 h at 37 ^o^C. When the incubation was longer than 2 h, PMSF was added again to the same final concentration. After that, the suspensions were centrifuged (10,750 x g for 1 min), the supernatants were kept at 4 ^o^C before further analysis, the cells were washed once and diluted to the same volume with 125 mM PPB pH 8.

### Solubilization of the dS and 3xL2-dS with detergent

After the treatment with enzyme and the following incubation in buffer, the cells were harvested by centrifugation and resuspended in PPB (pH 8) with concentrations between 50 and 250 mM. Then, 10% v/v Tween 20 or 10% v/v Triton X-100 (stock solutions in buffer) was added to final concentrations between 0.25% and 1%, and the suspensions were incubated under various conditions. The temperature of incubation was varied between 24 ^o^C and 45 ^o^C, and the period of incubation between 30 min and 4 h. To study the effect of NaCl on extraction efficiency, NaCl was added to the buffers to a final concentration of 150 mM. After appropriate periods of incubation with detergent, the cell suspensions were centrifuged at 10,750 x g for 5 min and the resulting supernatants were kept at 4 ^o^C before further analysis. Optionally, the insoluble fractions obtained were washed once with 125 mM PPB pH 8, diluted in the same buffer and analyzed for the recombinant proteins by SDS PAGE and Western blot.

The removal of Triton X-100 was carried out by batch incubation of the soluble fractions with Amberlite XAD4 beads for 2 h at room temperature as described by Kee et al. 2010 [14]. The amounts of Amberlite XAD4 required to achieve effective detergent removal was calculated on the base of the Product information sheet (Sigma).

### Preparation of the cell lysates

1. To determine the maximum expected protein content of the soluble cell protein, a 1 mL suspension untreated was centrifuged (10,750 x g, 1 min), the cells were diluted in 125 mM PPB (pH 8) to a final concentration corresponding to 180 mg wet cell weight mL^-1^ and DTT was added to final concentration of 5 mM. After 1 h of incubation at room temperature, the suspensions were mixed with an equal volume of glass beads (diameter 0.42–0.6 mm) and vortexed 10 times for 1 min each, with 30-s pause intervals with ice incubation, respectively. This procedure led to about 99% cell lysis, as determined by counting the cells in a Thoma chamber. The resulting lysate was centrifuged at 10 750 x g for 5 min, and the supernatant was kept at 4 ^o^C until total protein determination and SDS PAGE analysis.
2. To compare the extraction efficiency of the recombinant proteins, yeast cells not exposed to electric treatment were diluted in 25 mM Na-phosphate buffer, 2 mM EDTA, pH 8.0. Tween 20 (10% v/v stock solution in buffer) was added to a final concentration of 0.5%, and PMSF to final concentration of 2 mM [42]. The cell suspension was mixed with an equal volume of glass beads and vortexed 10 times for 1 min each with 30-s pause intervals with ice incubation, respectively. The homogenates were kept overnight (ON) at 4 ^o^C and after that clarified by centrifugation for 20 min at 10 750 x g, and the supernatant was kept at 4 ^o^C until further analysis.

### Analytical methods

Total protein content was determined according to Bradford [48] with bovine serum albumin as a standard.

Sodium dodecyl sulfate polyacrylamide gel electrophoresis (SDS-PAGE) of the protein samples was performed on 11% polyacrylamide slab gels as described by Laemmli [49]. Protein was detected by silver staining as described by Nesterenko et al. [50], and the protein molecular weight markers used were PageRuler™ Prestained Protein Ladder (10–170 kDa). Image analysis of each gel was performed by scanning with a PC scanner (UMAX Astra 600s) and saving in TIFF format. Densitometric analyses of gels images were performed with ImageJ. For Western blot analysis, the proteins were transferred onto nitrocellulose membranes by semi dry blotting according to Bjerrum and Schafer-Nielsen [51]. After blocking of the membranes, the anti-dS mouse monoclonal antibody (7C12) which detects the wild-type dS and the dS domain of the fusion proteins was used as a primary antibody [52, 53]. The detection was completed with AP-conjugated goat-anti-mouse antibody and Nitro blue tetrazolium /5-Bromo-4-chloro-3-indolyl phosphate (NBT/BCIP) solution.

Particle size distribution of VLP preparations was analyzed by dynamic light scattering using a Nanotrac Wave Particle Size and ZetaPotential Analyzer (Microtrac).

The yeast biomass was measured as dry cell weight (DCW) using an infrared moisture analyzer method according to Li and Mira de Orduña [54].

The results from three to six independent experiments are shown as a mean value ± standard deviation.

## Results and discussion

### Permeabilization of the cell envelope

To achieve a selective and efficient extraction of both recombinant proteins, the first step was to permeabilize irreversibly the plasma membrane and to increase the cell wall porosity by PEF treatment and a subsequent incubation of the cells with lytic enzyme. The aim was to remove at least part of the host soluble proteins, as well as other soluble contaminants. Since both recombinant proteins are integral membrane proteins, it was expected that they will be retained inside at this stage.

#### PEF treatment

The permeabilization of the plasma membrane was performed as the cell suspensions were treated with pulsed electric field at a flow rate of 25 mL min^− 1^. During their passage through the pulsing chamber, the cells received 30 pulses of 0.7 ms duration, and 41.7 Hz frequency. At these conditions, the residence time of the cells in the chamber was 0.72 s. Optimization of the electropulsation protocol was performed by varying the field strength only. The degree of the irreversible permeabilization was determined by loading the cells with PI, as described in Material and methods. Irreversible permeabilization of 85-99% of the treated cells was obtained at a field strength in the range of 5.5–5.85 kV cm^− 1^. Under these treatment conditions, the outlet temperature of the cell suspensions, measured 4–6 s after their passage through the pulsing chamber, was in the range of 58–62 ^o^С. The temperature of the collected samples incubated at room temperature dropped quickly (about 1 min) to 48–50 ^o^C.

#### Treatment with enzyme

To enhance the cell wall porosity, the cells were incubated with lyticase from *Arthrobacter luteus* and dithiothreitol (DTT) which breaks the disulfide bonds between the mannoproteins in the outer layer of the yeast cell wall. The goal was to facilitate the release of soluble proteins without causing significant cell lysis. Our expectation was that this treatment would also facilitate the passage of detergent micelles through the cell wall and the subsequent release of protein-detergent complexes into the environment. The effect of different concentrations of lyticase (2.5–30 U mL^− 1^) and DTT (1–10 mM) on the amount of released protein was studied.

**Fig. 1:**
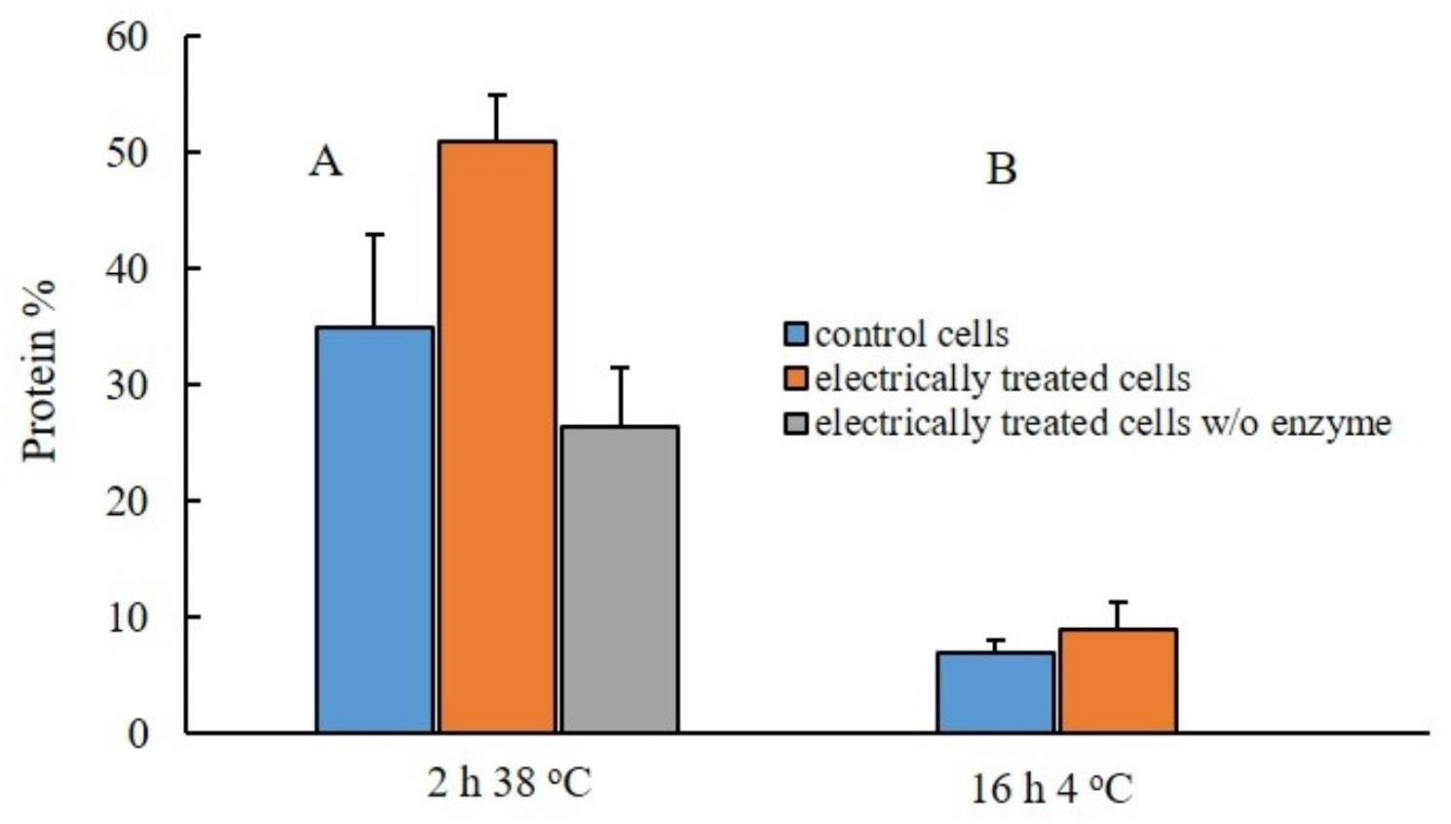
Protein release from control and electrically treated cells. (**A**) after incubation for 2 h at 38 °C with 20 U mL^− 1^ lyticase/5 mM DTT or only with 5 mM DTT; (**B**) after subsequent incubation for 16 h at 4 °C in 125 mM PPB pH 8. The values represent the mean ± SD of five different experiments

It was found that incubation of the electrically treated cells with 20 U mL^− 1^ enzyme and 5 mM DTT for 2 h is optimal for the subsequent extraction of the recombinant proteins with detergent. As shown in Fig. 1, under these incubation conditions, the electrically treated cells released about 50.9 ± 4% from the total soluble protein. In comparison, the protein released from cells incubated only with DTT was 26.4 ± 5%. The control cells (cells not treated with PEF) released about 35% from the soluble protein during the incubation with enzyme, which is significantly higher, than that obtained with another recombinant *H. polymorpha* strain earlier [40]. As the cells used in this study have been kept frozen before the procedure of extraction, this difference could be attributed to destabilization of the cell membranes during the process of freeze and thaw.

The release of proteins from electropermeabilzied yeast cells is a relatively slow process and depends on the temperature [32, 40]. Therefore, after incubation with the enzyme, the cells were washed, diluted to the same volume in 125 mM PPB pH = 8 containing 1.5 mM PMSF and incubated further for 1–3 h at room temperature or up to 48 h at 4 ^o^C. As shown in Fig. 1, during this second incubation, although slowly, soluble protein continued to be released from the cells.

### Detergent treatment

For the extraction of recombinant viral proteins, a solubilization with Тriton X-100 or Тween 20 is most frequently applied [10, 14, 16, 55].

Hence, firstly we used these two detergents to study the efficacy of the recombinant proteins release from the permeabilized cells. Triton X-100 and Tween 20 were added to the cell suspensions to final concentrations between 0.25% and 1%. To evaluate the optimal recovery conditions, the incubation temperature was varied between 24 ^o^C and 45 ^o^C, and the incubation time was between 30 min and 4 h.

**Fig. 2:**
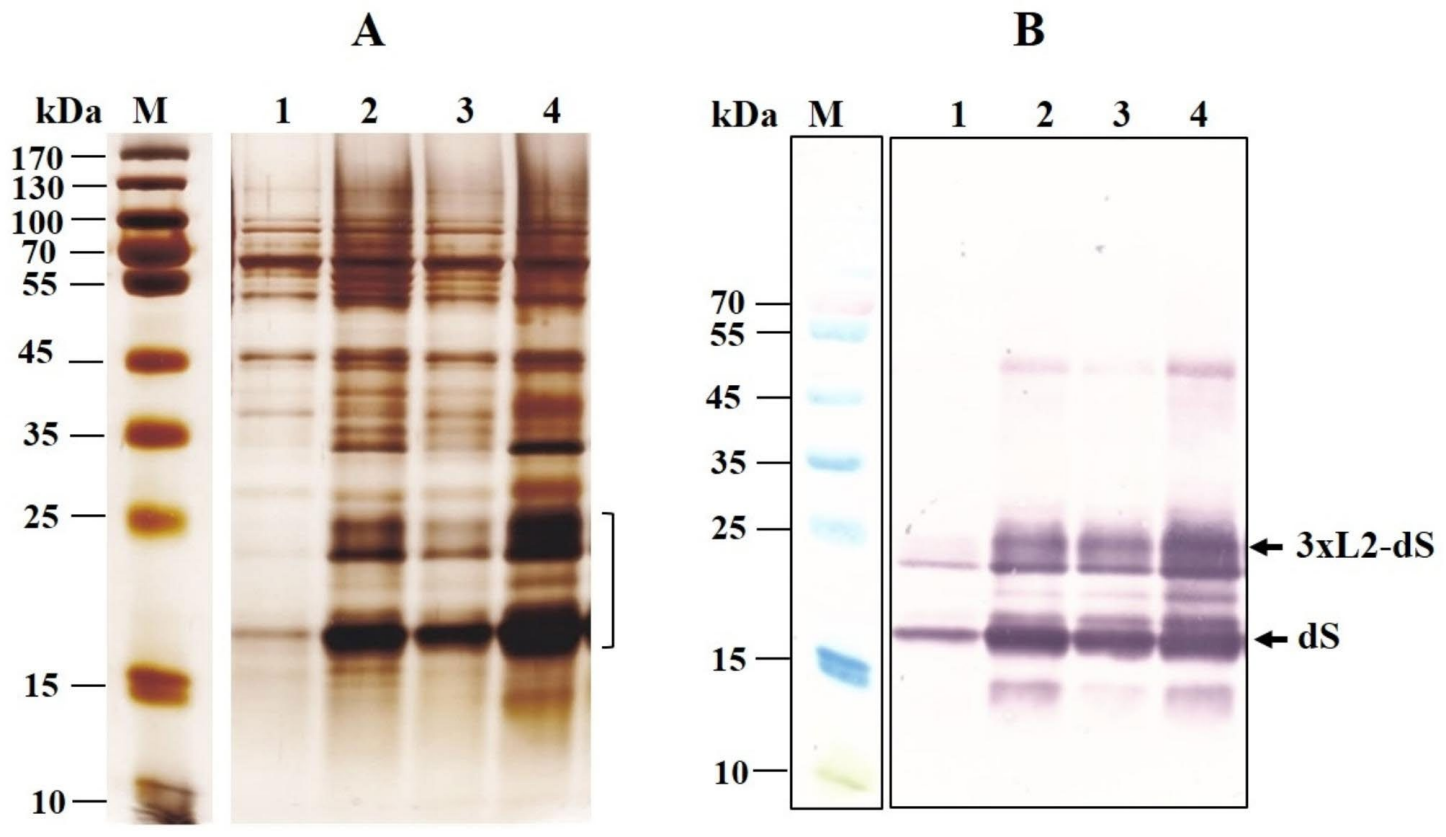
SDS PAGE (**А**) and Western blot (**B**) analysis of extracts from electrically treated cells obtained after 75 min incubation with 0.5% Tween 20 or 0.5% Triton X-100. Lane M, molecular weight marker; lane 1, soluble fraction obtained after incubation of the cell suspension (125 mM PPB, pH 8) for 75 min with 0.5% Tween 20 at room temperature; lane 2, 0.5% Тriton X-100 at room temperature; lane 3, 0.5% Tween 20 at 39 ^o^C; lane 4, 0.5% Тriton X-100 at 39 ^o^C. The same volumes of extracts obtained from cell suspensions with equal cell concentrations are loaded on to each lane

The SDS PAGE of the obtained extracts revealed the appearance of very strong bands after incubation with Triton X-100 (Fig. 2A, lanes 2 and 4). There was one band with a molecular weight (MW) of around 18 kDa, which corresponds to the theoretical MW of the dS monomer, and there were two bands very close to each other with a MW about 24 kDa, coresponding to the theoretical size of the fusion protein monomer. The Western blot analysis confirmed that these bands, along with a small band located in between, corresponded to the recombinant proteins. Two more bands were also observed: one with a MW of about 50 kDa, which probably corresponds to the oligomeric form of dS [10], and a small band with MW under 15 kDa. However, since they were not visible after silver staining of the gels, in the further analyzes of the extraction efficiency, the bands localized in the range 18–25 kDa were considered as the total amount of isolated recombinant proteins.

The data presented in Fig. 2 show significantly better release of the recombinant proteins upon incubation of the electrically treated cells with Triton X-100, then with Tween 20. The incubation temperature was also an important factor for efficient extraction. Optimal results were obtained at temperatures 39–42 ^o^C with incubation time of 75–90 min. It was found, that NaCl increases the extraction efficiency when using 50 mM PPB pH 8, but had no effect when detergent treatment was performed in 125 mM PPB. On the other hand, incubation of the cells in buffer with higher concentration (higher than 125 mM) did not improve the extraction efficiency (data not shown). Therefore, in all further experiments, the treatment with Triton X-100 was carried out in 125 mM PPB pH 8.

**Fig. 3:**
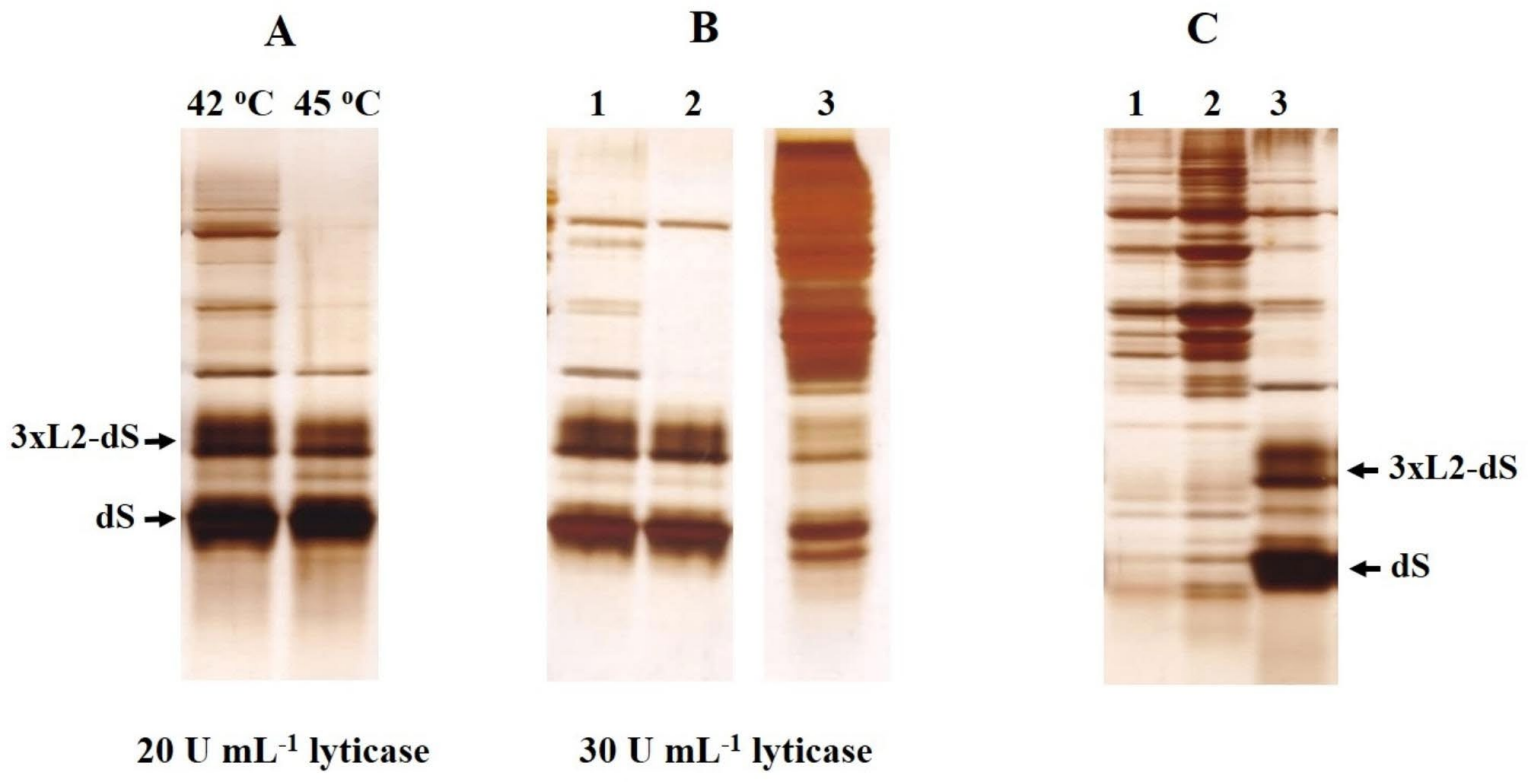
SDS PAGE analysis of: (**A)** extracts obtained after incubation of PEF treated cells with 0.5% Triton X-100 for 75 min at 42 ^o^C and 45 ^o^C; (**B**) soluble fractions (lanes 1 and 2) and insoluble fraction (lane 3) obtained after treatment of electropermeabilized cells preincubated with 30 U mL^− 1^ lyticase for 3 h at 42 °C with 0.5% Triton X-100. Lane 2 presents the same extract (lane 1) after incubation for 10 min at 60 ^o^C and centrifugation to remove the denaturated proteins; (**C**) soluble fractions obtained after treatment of cell suspensions (125 mM PPB pH 8) with 0.5% Triton X-100 for 75 min at 42 ^o^C: lane1, electropermeabilized cells preincubated for 2 h with 5 mM DTT; lane 2, control cells preincubated for 2 h with 5 mM DTT; lane 3, electropermeabilized cells preincubated for 2 h with 20 U mL^− 1^ lyticase/5 mM DTT

Changing the incubation temperature from 39 ^o^C to 42 ^o^C led to a further decrease of the host proteins in the extracts (Fig. 3A). The protein content of the extracts obtained after incubation with 0.5% Triton X-100 at 39–42 ^o^C for 75 min was 40.12 ± 2.2 mg g^− 1^ DCW. According to the analysis of the densitograms, the recombinant viral proteins purity was 53.9 ± 7%.

At higher incubation temperatures (45 ^o^C) most of the host proteins were eliminated, but there was also a decrease in the quantity of the extracted recombinant proteins.

As shown in Fig. 3B, part of the recombinant proteins remained in the insoluble fraction even when the cells were incubated with a higher enzyme concentration (30 U mL^− 1^) before Triton X-100 treatment. Comparison of band intensities (lanes 1 and 3) suggests that the extraction efficiency of the fusion protein is better than that of dS.

The increase in cell wall porosity, triggered by the enzyme, was critical for the release of the recombinant proteins (Fig. 3C). When cells were preincubated in 125 mM PPB pH 8 containing only DTT (lanes 1 and 2), during the following incubation with Triton X-100, they released numerous host proteins but not the recombinant proteins. As known, at concentrations above the critical micellization concentration (CMC) the detergents exist under form of micelles and monomers, which are in equilibrium. The hydrodynamic radius of Triton X-100 micelles is in the range of 4.18–5.57 nm in dependence on the temperature [56]. According to the proposed mechanisms, the species leading to membrane solubilization and extraction of integral membrane proteins can be the detergent monomers, the detergent micelles or both species interacting with the membranes in a different way [57–59]. One could assume that only the monomers of Triton X-100 could reach the membrane of the cells not treated with lyticase. The absence of recombinant proteins in the extracts obtained from these cells may be due to the inability of the detergent micelles to pass through the cell wall and/or to the retention of the formed detergent-protein complexes inside.

### Purification by heat treatment

**Fig. 4:**
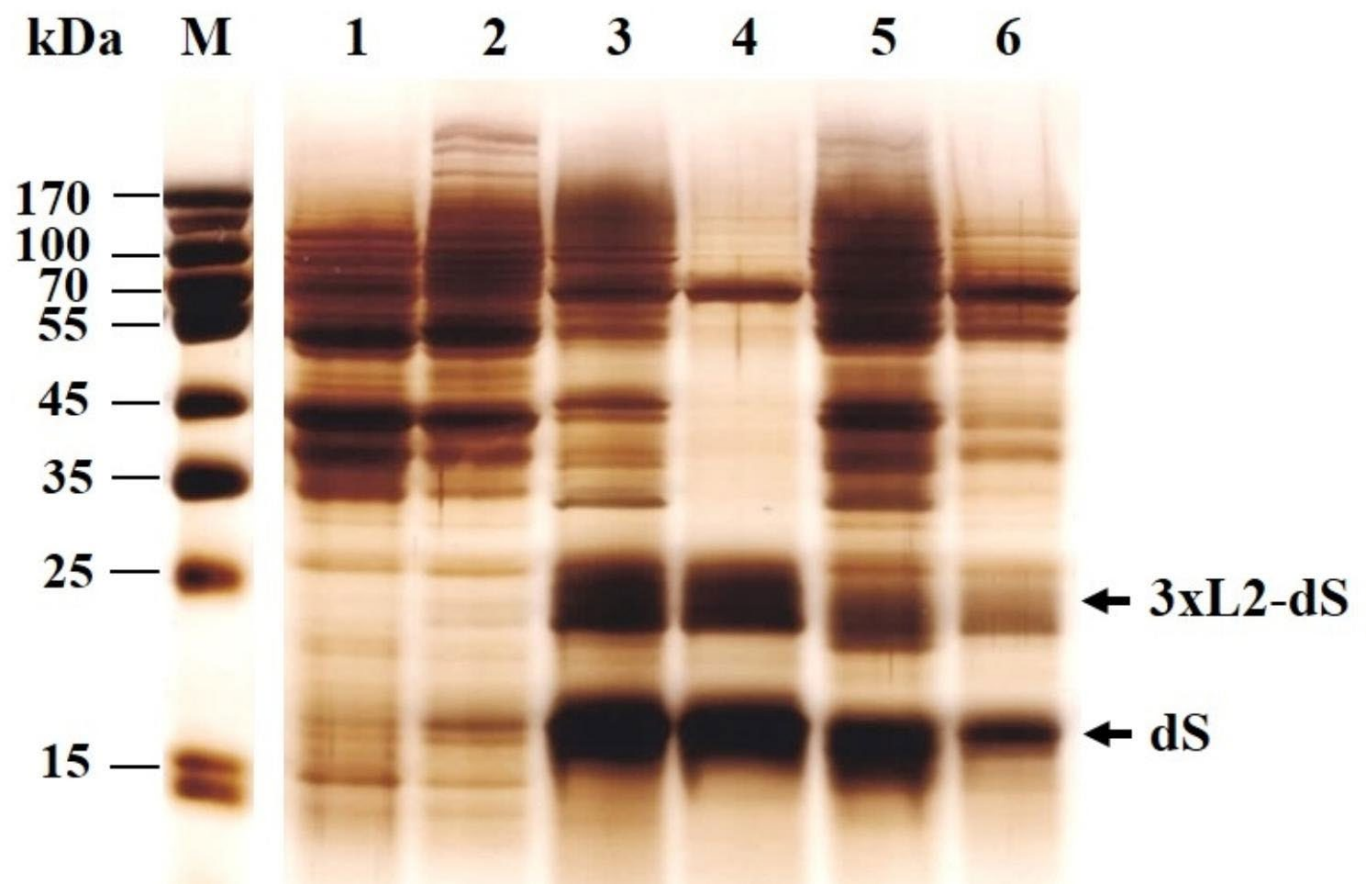
SDS PAGE analysis of the proteins released after incubation with lyticase and after the following treatment with detergent. Lane M, molecular weght marker; lane 1, proteins released from PEF treated cells after 2 h incubation in 125 mM PPB pH 8 with 20 U mL^− 1^ lyticase and 5 mM DTT; 2, proteins released from control cells under the same conditions of incubation; 3, proteins released after incubation of PEF treated cell with 0.5% Triton X-100 (75 min, 39 ^o^C); 4, the same extract as in 3 after 10 min incubation at 60 ^o^C; 5, proteins released from control cells incubated with 0.5% Triton X-100 (75 min, 39 ^o^C); 6, the same extract as in 5 after 10 min incubation at 60 ^o^C

Although part of the soluble proteins was released during and after the incubation with lyticase, the detergent treatment led to the presence of a few native proteins also. Comparing the protein profiles of the extracts obtained after enzyme treatment (Fig. 4, lanes 1 and 2) and after detergent treatment (Fig. 4, lanes 3 and 5), it seems that many soluble host proteins are released at this stage, too, together with the membrane proteins.

Precipitation of the host proteins by heat treatment is a simple and cheap method for partial purification of recombinant proteins that have high thermal stability [60–62]. In this study, to test the possibility for host proteins removal by heat treatment, two different approaches were applied. Firstly, the soluble fractions obtained after treatment with Triton X-100 were incubated at 60 ^o^C for various time intervals (10–30 min), after which the suspensions were centrifuged for 5 min at 10,750 x g to remove denatured proteins. The total protein in the supernatants was determined using the Bradford assay and the profile of the proteins was analyzed by SDS PAGE. As shown in Fig. 4 (lane 4), the incubation of the extracts obtained from PEF treated cells for 10 min at 60 °C led to precipitation of most of the contaminant proteins. A highly pronounced band corresponding to a host protein with a MW around 70 kDa is visible. As a similar band also appears in the extracts obtained after enzyme treatment (i.e. before incubation with Triton X-100), one could assume that this is a highly abundant, thermostable, soluble host protein.This brief heating did not significantly affect the amount of the recombinant proteins and the resulting purity on the protein level was 79 ± 3.2%. Longer incubation at 60 °C did not improve the purity; on the other hand, it reduced the recovery yield of both recombinant proteins (data not shown).

The treatment with Triton X-100 led to some liberation of recombinant proteins also from the control (PEF not treated) cells, however the heating of these extracts, resulted in a significant precipitation of the recombinant proteins (Fig. 4, lanes 5 and 6). Further studies are necessary to investigate the reason for the different termal stability of the recombinant proteins isolated from control and electropermeabilized cells.

In another set of experiments, thermal treatment of the cell suspensions was applied prior to the addition of Triton X-100. The idea was to induce denaturation and aggregation of the native soluble proteins that remained inside the cells after the enzyme treatment, so that they would be trapped in the cell wall. On the other hand, since the recombinant proteins are still inserted into the host membranes, conformational changes that would affect their immunogenicity are less likely to occur.

After incubation with lyticase and DTT, the cells were washed, diluted in 125 mM PPB pH 8 to the same volume, and incubated at 60 ^o^C between 10 and 30 min. Then Triton X-100 was added to a final concentration of 0.5% and the suspensions were further incubated at 39 ^o^C (in some experiments at 42 ^o^C) or at room temperature for 75 min.

**Fig. 5:**
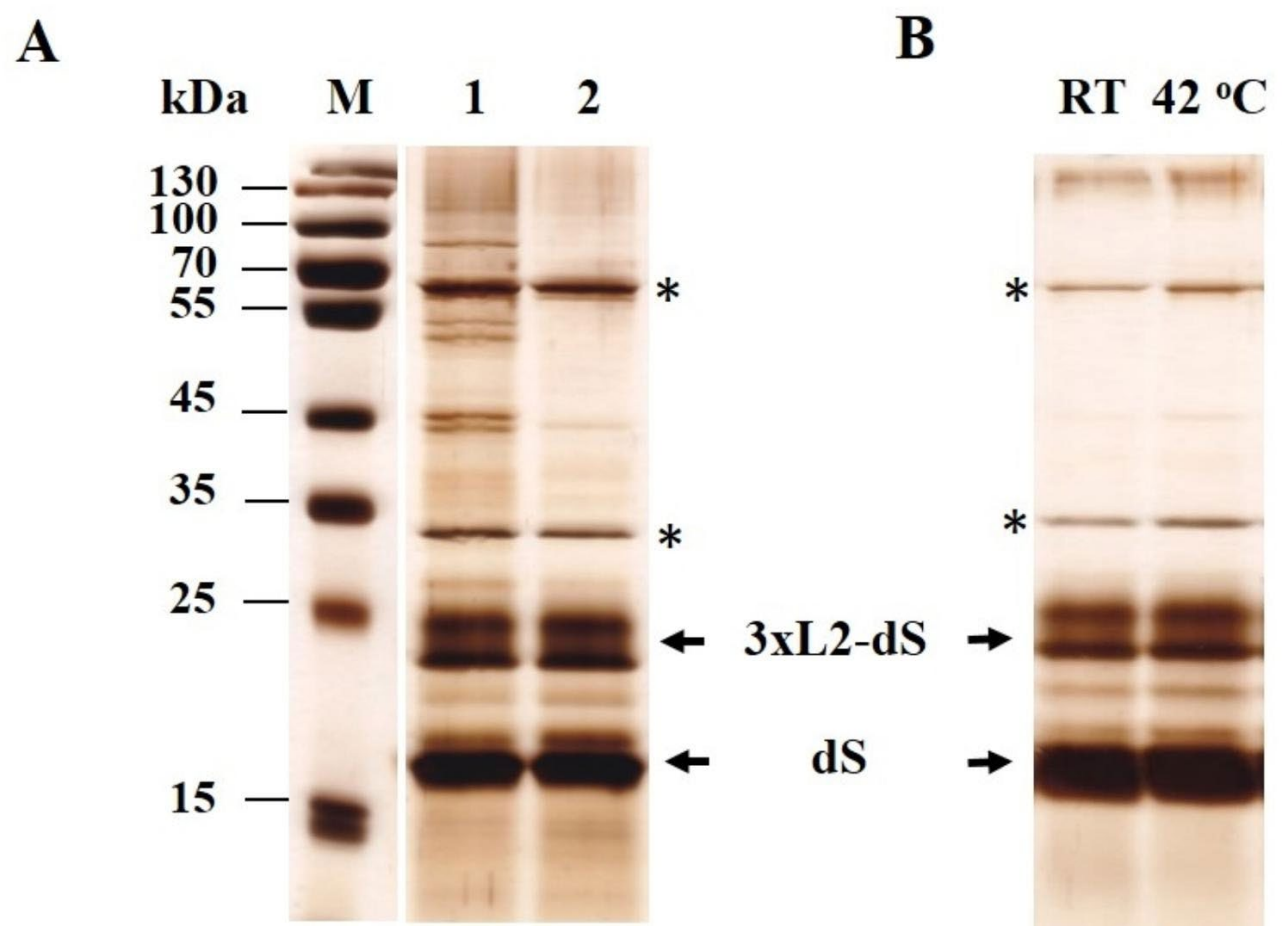
Effect of the preincubation of the cell suspension at 60 ^o^C on the extraction efficiency. (**A**) Lane M, molecular weight marker; lane 1, extracts obtained after incubation with 0.5% Triton X-100 for 75 min at 42 ^o^C; lane 2, extracts obtained from cells preincubated for 10 min at 60 ^o^C before Triton X-100 addition (0.5% Triton Х-100, 75 min at 42 ^o^C); (**B**) extracts obtained from cells preincubated for 10 min at 60 ^o^C and after that incubated with Triton X-100 at room temperature or at 42 ^o^C. The host proteins are indicated with asterisk

As shown in Fig. 5A, the preincubation of the cell suspensions for 10 min at 60 ^o^C, although not so efficient as the heating after Triton X-100 treatment, also led to a significant decrease of the host proteins. The extended incubation at 60 °C before detergent addition did not lead to better results (data not shown). The protein content of the extracts obtained was 27.8 ± 2.4 mg g^− 1^ DCW, and the purity on protein level was 71.4 ± 6.3%.

A heat treatment has been used for partial purification of VLPs forming recombinant hepatitis B virus core protein expressed in *E. coli* and yeast [63–65]. Li et al. [65] applied a two-step heat treatment for purification of HBc particles expressed in *E coli.* In this study, the soluble fraction obtained after cell disruption has been heated at 60 ^o^C for 30 min and then at 70 ^o^C for another 30 min, which resulted in 86% particle recovery and 75% purity, without affecting the immunogenicity of VLPs obtained. The same procedure has been successfully applied also for purification of HBc-derivative products containing epitopes from three different proteins. Freivalds et al. [64] incubated the clarified lysates of *P. pastoris* expressing recombinant hepatitis B virus core protein (HBc) for 1 h at 65 ^o^C. This treatment eliminated part of the host proteins but also the degraded forms of HBc protein.

In our system, most probably due to the more selective recovery of the recombinant proteins, similar degree of purification was obtained by a single and relatively short thermal treatment. Despite being less efficient for removing of the host proteins, the heating of the cells before Triton X-100 addition has had another positive effect. As indicated above (Fig. 2), the incubation of the cells with Triton X-100 at room temperature, led to lower extraction efficiency as that obtained by incubation at 39–42 ^o^C. However, when the cells were preincubated for 10 min at 60 °C before Triton X-100 addition, the extraction efficiency at room temperature becomes almost the same as the one obtained by incubation with Triton X-100 at 39–42 °C (Fig. 5B). Probably, the preincubation at 60 ^o^C provokes some changes in the mannoprotein layer of the cell wall, so that during the following incubation with Triton X-100 an additional increase of the cell wall porosity occurs.

### Treatment with KSCN

Although the heating of the cell suspensions before Triton X-100 addition led to a significant reduction of the host proteins, the SDS PAGE analysis revealed 2–3 highly pronounced bands corresponding to host proteins, which are obviously thermostable (Fig. 5).

A widely used approach for partial purification of recombinant proteins including recombinant hepatitis B surface antigen is the treatment with the chaotropic agent KSCN [66–68]. The choice to attempt further purification by using this compound was dictated also by additional considerations. According to the literature data, treatment of *S. cerevisiae* derived *HBsAg* particles with KSCN promotes VLPs maturation by formation of inter- and intramolecular disulfide bonds [55, 69, 70]. It was also shown that the final treatment of the HBsAg VLPs obtained from *P. pastoris* with 1.2 M KSCN at 37 °C, followed by dialysis against PBS, improved the fine-structure of the VLP surface and increased the number of VLPs with uniform size. [16, 71].

Considering these observations, we assumed that treatment with a chaotropic agent could be used to remove the thermostable host proteins, without risk of damaging the recombinant proteins. In this study, the cell suspensions were preincubated for 10 min at 60 ^o^C. After addition of Triton X-100 (0.5% final concentration) the suspensions were incubated at 39 ^o^C or at room temperature for 75 min. To remove the detergent, the extracts obtained were incubated with Amberlite XAD4 for 2 h at room temperature. After removal of the beads, KSCN was added to final concentration of 1.2 M, and the extracts were incubated at 37 ^o^C for 3 h. Next, they were centrifuged for 5 min at 10 750 x g, the supernatants obtained were dialyzed against phosphate buffered saline (PBS) pH 7.2 for 24 h at 4 ^o^C and stored at 4 ^o^C until SDS PAGE and Western blot analyses.

**Fig. 6:**
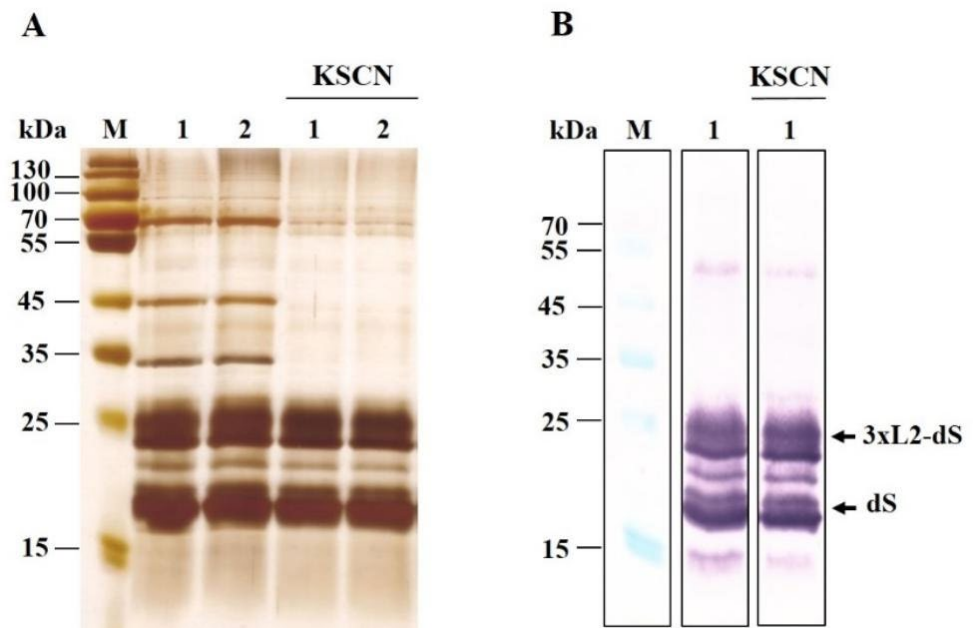
SDS PAGE (**A**) and Western blot (**B**) analysis of the proteins extracted from PEF treated cells before and after XAD 4 and KSCN treatment. Lane M, molecular weght marker; lane 1, extracts from cells incubated with 0.5% Triton X-100 for 75 min at 39 ^o^C; lane 2, extracts from cells incubated with 0.5% Triton X-100 at room temperature

The SDS PAGE analysis demonstrated that the incubation with KSCN led to the removal of most of the host proteins from the extracts (Fig. 6).

The recombinant proteins were largely resistant to this treatment. The protein content of the extracts obtained after KSCN treatment was 18.1 ± 2.3 mg g ^− 1^ DCW. According to the analysis of the densitograms, the VLPs purity on protein level was 80 ± 4.6%.

**Fig. 7:**
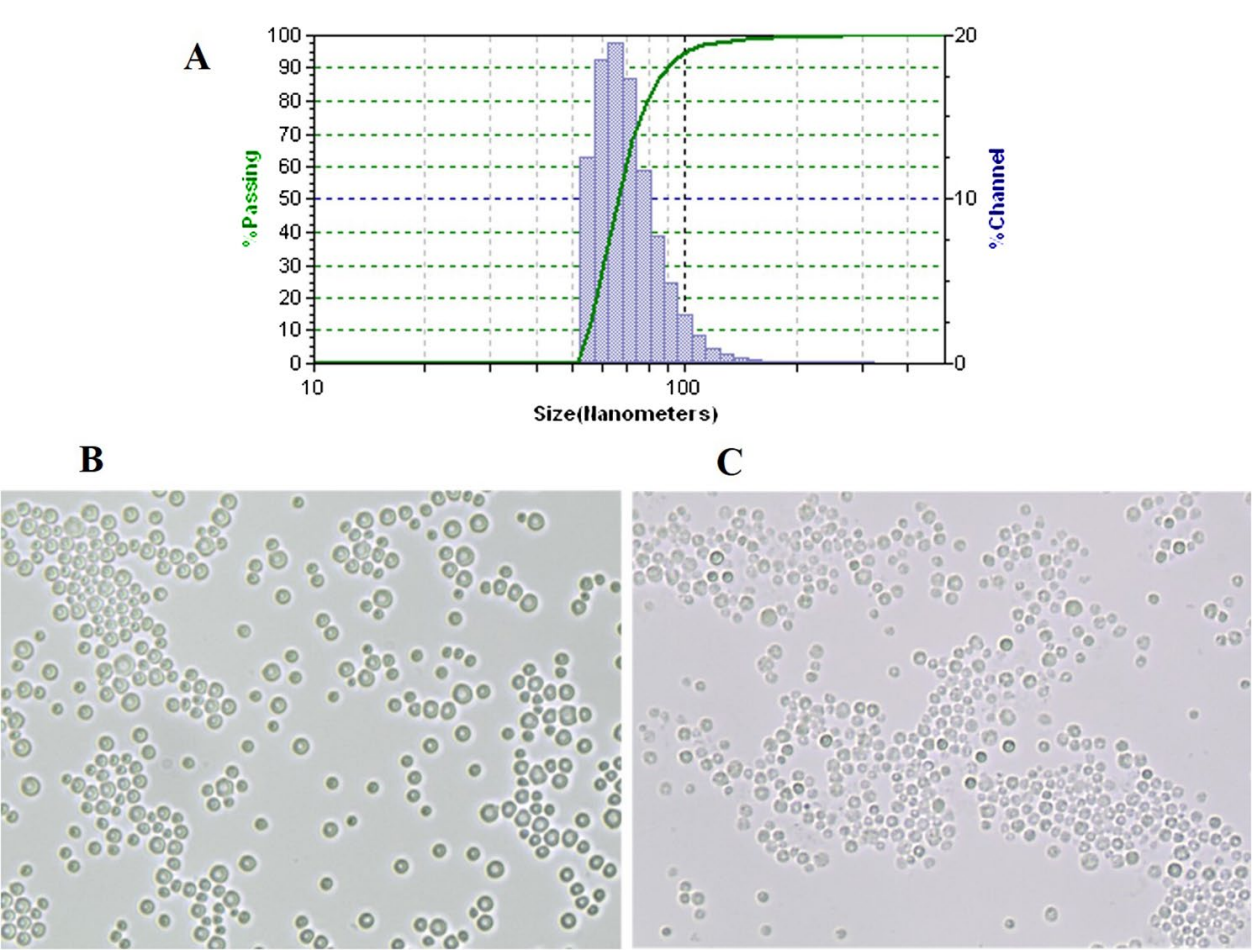
Dynamic light scattering measurements of particle sizes (**A**). Light micrographs of electropermeabilzied cells before (**B**) and after (**C**) treatment with enzyme and extraction of the recombinant proteins with 0.5% Triton X-100 at 39 ^o^C

The analysis of the size of the formed chimeric VLPs was performed by Dynamic light scattering after filter sterilization of the samples and their storage for 3 weeks at 4 ^o^C. About 80% of the particles have diameters in the range of 43–79 nm, and the pick mean diameter was 55.2 ± 7.8 nm (Fig. 7A).

After the incubation with enzyme and the following extraction with Triton X-100, most of the cells retained their integrity, which facilitates their removal after the release of the recombinant proteins (Fig. 7B and C).

### Extraction of recombinant proteins from strain T#3–3

To assess the applicability of the developed protocol for recovery of integral membrane proteins from other strains of *H. polymorpha*, some of the experiments described above were repeated with strain T#3-3 co-expressing dS and the fusion protein EDIIIWNV-dS. The electrical treatment was performed under conditions similar to those applied to strain #25-5. The temperature rise during pulse application ranged from 45 to 57 °C, and the extraction process was studied with cell suspensions where more than 80% of the cells were irreversibly permeabilized. The first experiments were conducted under conditions optimal for the recovery of dS and 3xL2-dS from strain #25- 5.The electrically treated and control cells diluted in 125 mM PPB pH 8, were incubated for 2 h with lyticase (final concentration between 10 and 20 U mL^− 1^) and 5 mM DTT to enhance cell wall porosity and to remove part of the soluble host proteins. Then, the cell suspensions were treated with Triton X-100 to solubilize the recombinant proteins. The incubation temperature ranged from 39 to 41 °C, the incubation time varied between 75 and 90 min, and Triton X-100 concentrations of 0.5% and 1% were tested.

**Fig. 8:**
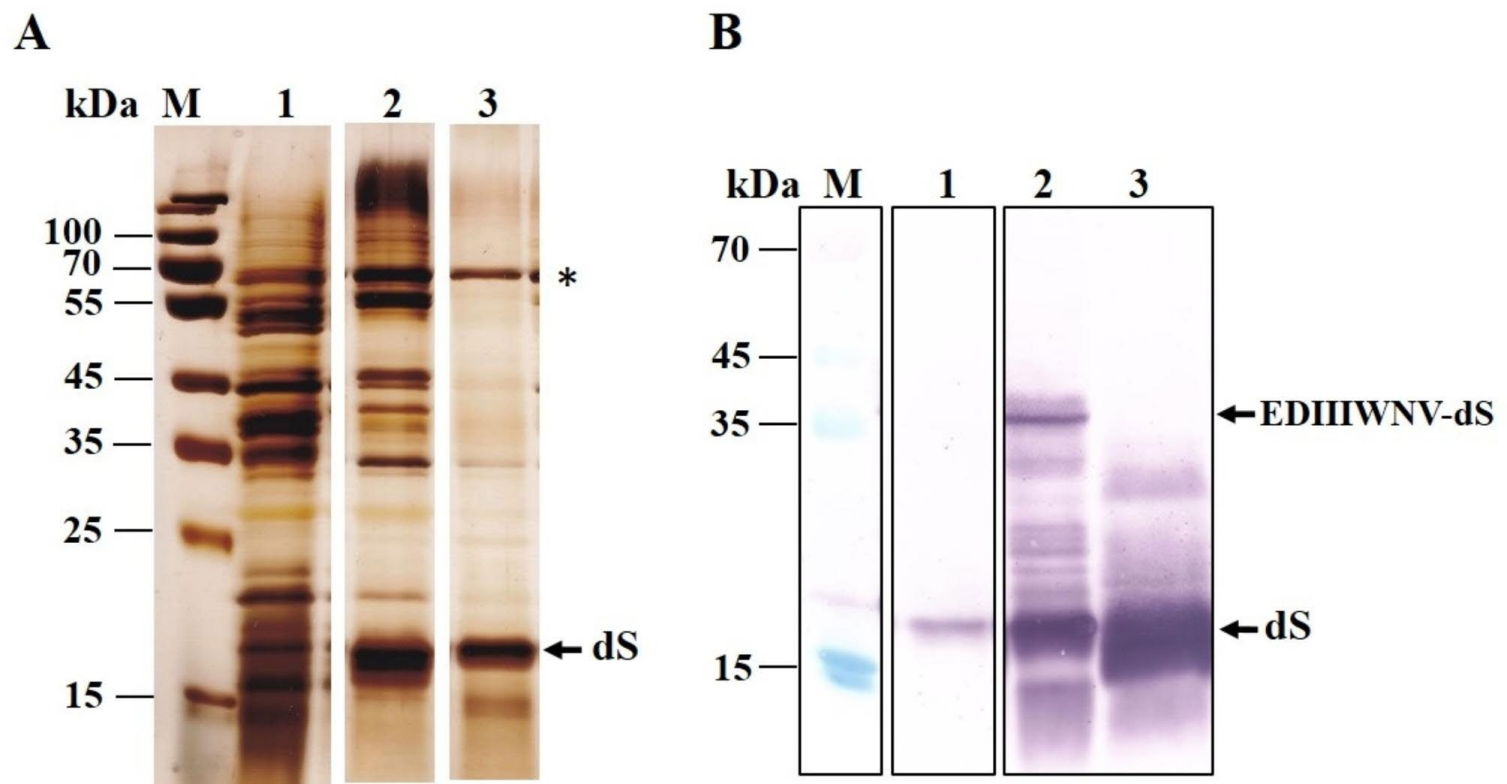
SDS PAGE (**A**) and Western blot (**B**) of the proteins released from PEF treated cells after incubation with lyticase and the following treatment with detergent. (**A**) Lane M, molecular weight marker; lane 1, proteins released from PEF treated cells after 2 h incubation with 20 U mL^− 1^ lyticase and 5 mM DTT; lane 2, proteins released during incubation with 1% Triton X-100 (75 min, 39 ^o^C); lane 3 extracts obtained from cells preincubated for 10 min at 60 ^o^C and subsequently incubated with Triton X-100 at 39 ^o^C. The thermostable host protein is indicated with asterisk. (**B**) Lane M, molecular weight marker; lane 1, supernatant obtained after treatment with enzyme; lane 2, insoluble fraction obtained after treatment with Triton X-100; lane 3, soluble fraction (A, lane 2) obatined after treatment with Triton X-100

**Fig. 9:**
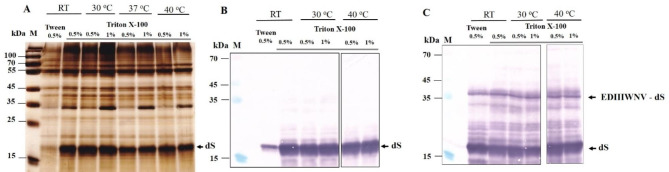
Analysis of the proteins in the soluble and insoluble fractions obtained after incubation of lysates with detergent for 90 min at different temperatures. (**A**) SDS PAGE of the proteins in the soluble fraction; (**B**) Western blot of the proteins in the soluble fractions; (**C**) Western blot of the proteins in the insoluble fractions

The SDS-PAGE (Fig. 8A) revealed a significant release of dS. Similar to strain #25-5, preheating for 10 min at 60 °C before the addition of Triton X-100 led to the removal of most host proteins, except for a thermostable, soluble host protein with a molecular weight of approximately 70 kDa. As demonstrated in experiments with strain #25-5, this protein can be denatured by incubating the extracts with KSCN. These preheating however, resulted also in about two-fold reduction in the amount of dS in the extracts, an effect that needs to be studied in more details. The Western blot analysis (Fig. 9B) showed that the enzyme treatment did not cause significant cell lysis, larger part of dS was released from the cells, but the fusion protein as well as a part of dS were retained inside.

The possible reasons could be, that the solubilization of the fusion protein EDIIIWNV-dS is not efficient under the applied conditions, or it is unstable and aggregates, preventing its passage through the cell wall. To distinguish between these two effects, electrically treated cells (temperature rise during pulse application about 50 ^o^C) were diluted in a hypotonic medium (50 mM PPB, pH 8) to a final concentration of 180 mg mL^− 1^ cell wet weight. Lyticase and DTT were added to final concentrations of 40 U mL^− 1^ and 5 mM respectively, and the suspensions were incubated at 38 ^o^C. To prevent proteolytic degradation, PMSF was added twice to the suspensions: before the addition of the enzyme and after 2 h of incubation, each time to a final concentration of 2 mM.

It was found that after 3 h of incubation with the enzyme, almost complete cell lysis occurred. The lysates were centrifuged for 15 min at 10 750 x g and washed with the same buffer to remove the soluble proteins. The pellets were gently resuspended in 125 mM PPB pH 8. Tween 20 was added to a final concentration of 0.5%, and Triton X-100 was added to a final concentrations of 0.5% and 1%. The suspensions were incubated for 90 min at different temperatures: room temperature (RT), 30 °C, 37 and 40 °C. Afterward, they were centrifuged for 10 min at 10 750 x g, and the proteins in the supernatant and the insoluble fractions were analyzed by SDS-PAGE and Western blot.

As shown in Fig. 9A, the solubilization of dS with 0.5% Tween 20 is not very efficient. On the other hand, Triton X-100 solubilizes dS relatively well even at room temperature. It seems that the most efficient solubilization occurs at 30 °C with 0.5% Triton X-100. Additional experiments have to be performed to refine the optimal temperature range for solubilization of dS. At 37 °C, the amount of the recombinant protein in the soluble fractions obtained after treatment with both concentrations of Triton X-100 decreases; most probably, part of the solubilized protein aggregates under these conditions and remains in the insoluble fraction. Western blot analysis of the soluble (Fig. 9B) and insoluble fractions (Fig. 9C) revealed that, under all tested conditions, the fusion protein as well as a part of dS remained in the insoluble fractions. Some of the multiple bands appearing between 15 and 35 kDa probably result from the partial proteolytic degradation of the fusion protein despite the addition of PMSF.

**Fig. 10:**
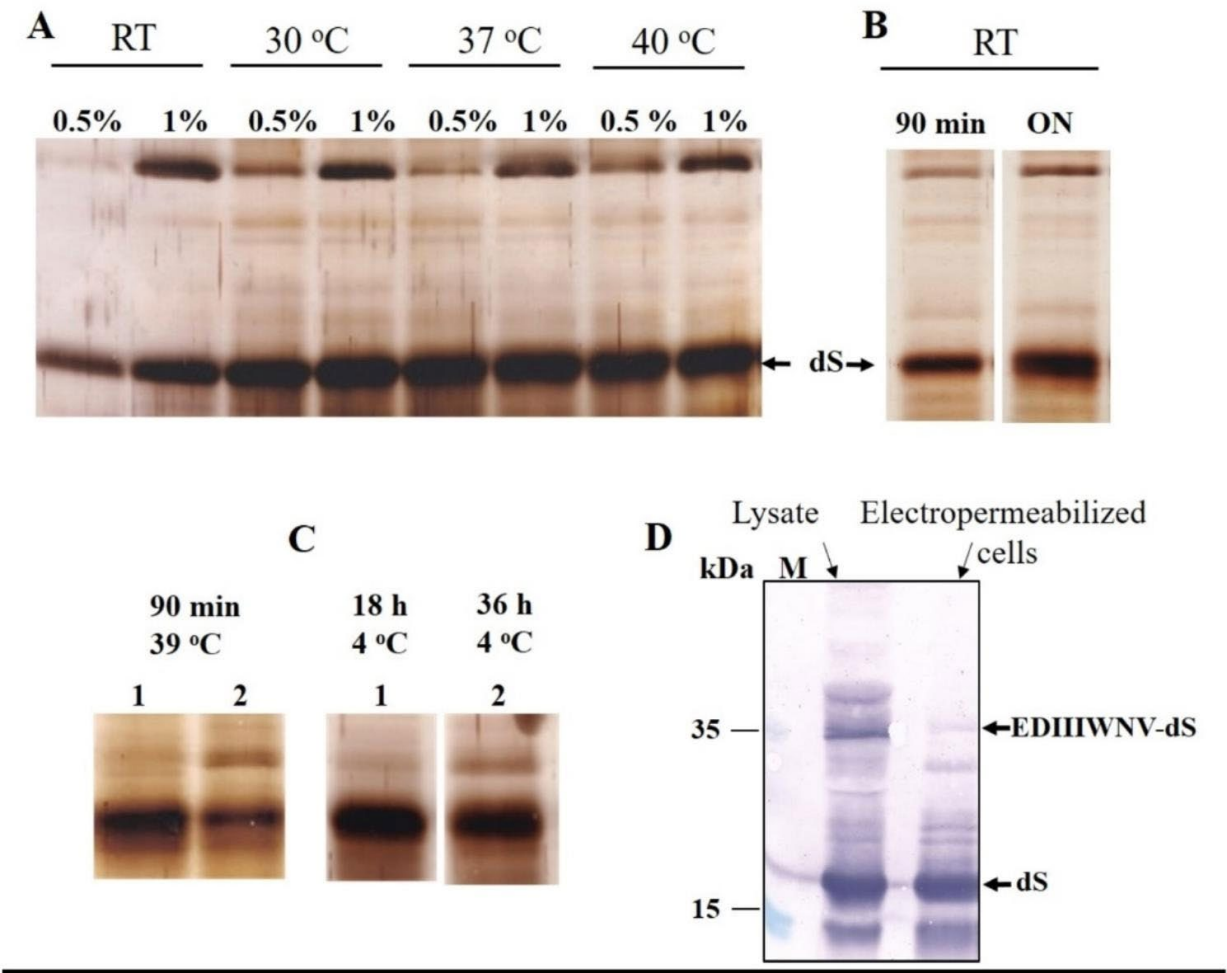
(**A**) SDS PAGE of the proteins released from PEF treated cells after 90 min incubation with Triton X-100 at different temperatures; (**B**) proteins released from PEF treated cells after incubation with 0.5% Triton X-100 at RT; (**C**) cComparrison of the yields of dS obtained from PEF treated (1) and control (2) cells under different conditions of incubation with 0.5% Triton X-100; (**D**) Western blot analysis of the soluble fractions obtained after mechanical disruption of the cells and ON incubation with Tween 20 at 4 ^o^C, and the proteins released from PEF treated cells after incubation with 0.5% Triton X-100, ON at 4 ^o^C

A similar experiment was conducted using electropermeabilized and control cells, preincubated with lyticase and DTT as already described. The cell suspensions were incubated with Triton X-100 for 90 min at different temperatures and the soluble fractions obtained were analysed by SDS PAGE (Fig. 10A). The analysis of the densitograms revealed that the optimal release of dS occurs in the temperature range between 30 and 37 °C. The protein content of the extracts obtained under these conditions was 31.4 ± 3.1 mg g^− 1^ DCW, with a purity on protein level of 25.2 ± 3%. Overnight incubation of the cells with 0.5% Triton X-100 at room temperature improved the release of dS (Fig. 10B). At more prolonged incubation (18–36 h; 4 ^o^C) the protein yield reached 40 ± 1.5 mg g^− 1^ DCW, but the purity decresed (15 ± 1.2%). Surprisingly, during prolonged incubation with detergent (up to 36 h at 4 °C), a significant amount of dS was released from control cells as well (Fig. 10C).

Western blot analysis revealed that the fusion protein appeared in the extract obtained from PEF treated cells after about 15 h of incubation with 0.5% Triton. However, even after incubation for 36 h at 4 ^o^C, the release efficiency was considerably lower, than what was achieved after mechanical disruption of the cells and overnight (ON) incubation with 0.5% Tween 20 (data not shown).

The results obtained so far lead to the conclusion, that the optimal conditions for the recovery of dS from both strains are different. This can be partly explained by differences in the cell wall structure. The optimal recovery of both recombinant proteins expressed in strain #25-5 was achieved by incubation with Triton X-100 at relatively high temperatures. Furthermore, the solubilized recombinant proteins exhibit a very high thermostability (Fig. 4). On the other hand, experiments with enzymatically lysed cells from strain T#3-3 demonstrate that even incubation with 0.5% Triton at 37 °C results in a decrease of the soluble dS, most probably due to denaturation, leading to its aggregation. Clearly, the same control experiments have to be conducted with strain #25-5 to clarify whether the optimal temperature for the solubilization of the recombinant proteins expressed in this strain is the same.

It is also unclear why the fusion protein 3xL2-dS releases almost completely in about 75 min from the cells of strain #25-5, as well as a part of dS expressed in both strains, while another part of this protein as well as the fusion protein EDIIIWNV-dS were retained within the cells, most probably because they cannot be solubilized, as shown with strain T#3-3. One can not exclude that the membrane domains where these proteins reside are too rigid to be solubilized under the conditions tested so far. Another possibility is that, due to inability to fold correctly, part of the expressed recombinant proteins are not membrane-bound proteins, but instead form aggregates (or structures) accumulating in different cellular compartments [15, 55, 72, 73]. The procedures applied for their solubilization differ significantly from the treatment found in this study as suitable for fast and efficient solubilization of a part of the recombinant proteins expressed in both strains, but also of many native membrane proteins (Fig. 9A).

The data obtained lead to the conclusion that the recovery of integral membrane proteins from permeabilized cells results from the interplay of different factors. This involves both, the efficiency of detergent-mediated solubilization and the stability of the solubilized protein under given conditions (time and temperature of incubation) on one hand, and the efficiency of the passage of the protein-detergent complexes through the cell wall on the other. It appears that heat treatment before detergent addition and relatively high temperatures during detergent treatment might lead to changes in the cell wall structure, which can facilitate the release of the solubilized proteins as long as they are stable and do not aggregate under the given conditions. With a high probability, this effect will depend on the structure of the cell wall and will vary among different strains. One may expect that the electroinduced changes in the cell wall structure, leading to higher sensitivity to lytic enzymes, will vary also depending on the yeast strain.

Clearly, further investigations need to be conducted with other systems - different *H. polymorpha* strains (but also other yeasts) expressing various recombinant integral membrane proteins to assess the effectiveness of the approach described in this article. It is possible that for some systems, this treatment may be highly efficient, as demonstrated here for the fusion protein 3xL2-dS and dS expressed in both strains, where the yields obtained significantly exceed those obtained by mechanical disruption of the cells. Furthermore, as demonstrated with strain #25-5, by using two simple procedures - brief heat treatment before detergent addition and incubation of the extracts obtained with 1.2 M KSCN one can achieve a protein purity of about 80%.

On the other hand, for other systhems, obviously, mechanical disruption in combination with detergent treatment will be more suitable.

If the method proves successful, as shown in the example of strain #25-5, PEF treatment applied in a flow mode is a scalable method suitable for processing of significant cell biomass [31, 74]. Irreversible permeabilization of yeasts leading to an efficient protein extraction can be obtained after a single passage of the cells through the pulsing chamber, where they receive a defined number (15–30) of electric field pulses with duration of 0.5-1 ms and moderate field strength (2–6 kV cm^− 1^).

The upscaling of PEF treatment can be done by increasing the flow rate and the biomass concentration. As shown recently, there is no significant change in PEF treatment efficiency even at flow rates of up to 130 mL min^− 1^. Additionally, it is possible to treat yeast cell biomass at concentrations of at least 85 g DCW L^− 1^ [32]. Data obtained from algae suggest that PEF efficiency remains unaffected even at biomass concentrations of 167 g DCW L^− 1^ [31.

PEF treatment is considered as a mild, non-thermal technique, as the irreversible plasma membrane permeabilization (i.e. loss of membrane barrier function) is a result of induction of an additional transmembrane potential. This is particularly important when the intracellular compound of interest is sensitive to temperature effects and mechanical stress (shear force or pressure changes) as the viral recombinant proteins forming eVLPs [75].

When very dense cell suspensions are exposed to electrical field pulses leading to irreversible permeabilization, a significant increase in temperature due to Joule heating is possible. This is a consequence of the strong increase in conductivity during pulse application, because of an instantaneous release of intracellular ions. As shown however, at electrical conditions leading to maximal protein release, no significant protein denaturation (loss of activity) takes place [32, 40]. According to the data obtained with different yeast species, including *H. polymorpha*, a suitable combination of electric field parameters, concentration, and output conductivity of the cell suspension allows the treatment to be conducted at room temperature without the need for a cooling system in the chamber.

The PEF treatment causes irreversible loss of plasma membrane integrity and enhances the cell wall porosity, but the release of both recombinant proteins was observed only after an additional increase of the cell wall porosity by incubation of the electropermeabilzed cells with lytic enzyme and thiol compound.

Lyticase is an expensive enzyme, and the concentrations used in this study are relatively high. However, different approaches could be applied to optimize this step of the purification procedure. The yeast cell wall porosity can vary significantly depending on the conditions of their cultivation [76]. A decrease in the optimal enzyme concentration could be achieved, for example, by using cells cultivated and induced at a lower temperature (30 °C), which leads to the development of more permeable cell walls. Another possibility could be the utilization of other, less expensive enzymes as demonstrated earlier [40, 41].

## Conclusions

This study demonstrates that the permeabilization of the cell envelope, achieved through subsequent pretreatment with a pulsed electric field and lytic enzyme, allows for the selective and high-yield recovery of both viral proteins expressed in *H. polymorpha* #25-5 as well as dS expressed in strain T#3–3 under conditions, suitable for their solubilization. The alternative approach for the isolation and partial purification of recombinant viral proteins described here has the potential to be applied to other strains *of H. polymorpha* expressing various recombinant membrane proteins.

## Data Availability

All data are included in the manuscript. Further queries or additional information can be requested to the corresponding author.
